# Bitter Melon (*Momordica charantia* L.) Fruit Bioactives Charantin and Vicine Potential for Diabetes Prophylaxis and Treatment

**DOI:** 10.3390/plants10040730

**Published:** 2021-04-08

**Authors:** Mahwish, Farhan Saeed, M. Tauseef Sultan, Ayesha Riaz, Sagheer Ahmed, Nicusor Bigiu, Ryszard Amarowicz, Rosana Manea

**Affiliations:** 1 Institute of Home & Food Sciences, Government College University, Faisalabad 38000, Pakistan; mahwishyounas@yahoo.com (M.); f.saeed@gcuf.edu.pk (F.S.); 2 Institute of Food Science and Nutrition, BZU, Multan 60800, Pakistan; tauseefsultan@bzu.edu.pk; 3 Institute of Home Sciences, University of Agriculture, Faisalabad 38000, Pakistan; i_sha_r@yahoo.com; 4 Shifa College of Pharmaceutical Sciences, Shifa Tameer-e-Millat University, Islamabad 44000, Pakistan; sagheer.scps@stmu.edu.pk; 5 Faculty of Medicine, Transilvania University of Brasov, 500019 Brasov, Romania; nicusorbigiu@yahoo.com (N.B.); rosanamanea@gmail.com (R.M.); 6 Institute of Animal Reproduction and Food Research, Polish Academy of Sciences, 10-748 Olsztyn, Poland

**Keywords:** bitter melon, *Momordica charantia*, diabetes, charantin, vicine, glycemic control, hypoglycemia

## Abstract

Natural products are gaining clinical significance in modern day health care systems to prevent diseases. Bitter melon, a health promoting vegetable, is traditionally used for medical nutrition therapy to cure diabetes but to reap maximum health claims, vigilant control of its substances in diet is crucial as part of curative action for effective diabetes management. In the present research, first phase focused on detection of key bioactive components, i.e., charantin and vicine in different parts of its fruit. In the second phase, normal and hyperglycemic Sprague Dawley rats were fed on skin, flesh and whole fruit of bitter melon at 150 and 300 mg/kg body weight and assessed for diabetes prophylaxis and treatment. The highest amount of charantin (0.16 ± 0.02 mg/g) was recorded in flesh while vicine was present in abundance in whole fruit (0.21 ± 0.01 μg/100 g). In normal rats, bitter melon supplementation was helpful in managing the onset of diabetes. Hyperglycemic rats showed diabetic complications including polydipsia, polyuria, glycosuria, renal hypertrophy and increased glomerular filtration rate. However, bitter melon consumption showed significant improvements in these parameters. The most potent dose was 300 mg/kg whole fruit that resulted in 31.64% lowering of blood glucose level and 27.35% increase in insulin level in hyperglycemic rats.

## 1. Introduction

Sedentary lifestyle and poor dietary habits lead towards occurrence of various disorders such as diabetes, cardiovascular diseases, hypertension, etc. Plants and their products have tendency to ameliorate such disorders and can play a helpful role in preventing diseases. The dietary components play an integral part in promoting the health due to presence of phytochemicals and phytonutrients. They are helpful in mitigating serious disorders such as diabetes, hypercholesterolemia, hyperlipidemia, inflammation, etc.

Bitter melon (*Momordica charantia* L.) is considered due to its chemical moieties [1]. The bitter melon is a member of family Cucurbitaceae and is also known as balsam pear, bitter gourd and karela in different regional languages. It is consumed as a vegetable in different tropical and subtropical regions of world, owing to its distinctive taste and nutritional profile. Green fruits of bitter melon contain vitamin A, C, thiamine, niacin, riboflavin and minerals [2]. It naturally possesses high phenolic content, i.e., gallic acid, alkaloids, saponins, flavonoids, etc. Due to this rich phytochemistry, bitter melon exhibits highest antioxidant activity among its family. Owing to the presence of various bioactive components, bitter melon holds some pharmacological properties and exerts various health enhancing properties such as scavenging of free radicals, hypoglycemic and hypolipidemic activities [3]. Diabetes mellitus is a growing threat to human health that affects millions of peoples each year. The dietary patterns significantly influence the pathogenesis of diabetes mellitus by incorporating healthy foods in normal diets along with physical activity is an effective strategy. Many studies suggest the effectiveness of bitter melon against diabetes prophylaxis [4,5,6,7,8,9]. Many in vivo studies confirmed the hypoglycemic potential of bitter melon and treatment due to the presence of numerous hypoglycemic agents such as alkaloids, flavonoids, saponin, catechins, charantin, vicine, and polypeptide-p fractions [10]. Many experiments supported that bitter gourd proved to be effective in insulin production and can be hepato-renal protective. However, inadequacies were present in most of the studies due to poor study design [11]. Furthermore, some hypoglycemic studies reported contradictory results with negligible positive effects of bitter melon intake during diabetes [12,13]. In many studies, the data were insufficient and indecisive to advocate its use in diabetes management [11,14]. Some studies evaluated effectiveness of only specific part of bitter melon with single dosage for limited time period [12]. In these studies, no information was provided in relation to role of bioactive molecules in diabetes management. It was, therefore, important to conduct a more conclusive and systematic study. Hence, present study was designed to compare different parts of bitter melon fruit for charantin and vicine contents and subsequent feeding trial to assess individual or combined role of these natural products in improving diabetes and associated complications.

## 2. Materials and Methods

### 2.1. Procurement and Handling of Raw Material

Bitter melon fruits were procured from Vegetable Research Section, Ayub Agriculture Research Institute, Faisalabad, Pakistan. These fruits were washed carefully to remove adhered unhygienic substances and other contaminants. After washing, the outer warty skin was peeled and collected separately. After peeling, flesh part was obtained by removing seeds. Some fruits were cut into small size as a whole. These isolated fruit portions were kept for few days at room temperature to dry. Fine powder was made of this dried material by using laboratory grinder (Panasonic, Osaka, Japan, Model MJ-W176P) and absolute refined powder was collected by passing through a sieve and finally packed separately in glass bottles for further evaluation.

### 2.2. Reagents and Standards

HPLC grade reagents and standards were purchased from Sigma-Aldrich (Tokyo, Japan) and Merck (Darmstadt, Germany). Diagnostic kits were purchased from Cayman Chemicals (Europe) and Sigma-Aldrich Bioassay (Darmstadt, Germany).

### 2.3. Quantification of Charantin

The estimation of charantin contents in samples were determined by protocol of Pitipanapong et al. [15]. Firstly, 1.0 g powder of bitter melon samples was taken to extract with ethanol and further extracted in methanol by ultra-sonication. In this way, viscous crude extract of charantin was obtained by filtration of extracts and subsequent evaporation. Before analyzed by HPLC, crude extract was purified. HPLC analysis was performed with C-18 Inertsil ODS-3 column (size of particles 5.0 μM, 4.6 mm × 250 mm ID). Methanol-water (100:2 *v*/*v*) was used as mobile phase with 1 mL/min flow rate. The wavelength for UV detection was 204 nm and volume for sample injection was 200 μL.

### 2.4. Quantification of Vicine

The extraction of bitter melon powder samples was done with water (10–25 mL) for ten min by ultra-sonication. The mixture was centrifuged and supernatant was collected separately in volumetric flask. All the samples were filtered before analysis through HPLC. HPLC analysis was performed with C-18 reversed phase column 250 mm × 4.6 mm, 5 μM). Methanol-phosphate buffer with pH 3.0 (10:90, *v*/*v*) was used as mobile phase. Detector was set at 280 nm [16].

### 2.5. Efficacy Study

To probe anti-diabetic effect of bitter melon, Sprague Dawley rats were kept in Animal Room, College of Pharmacy, Government College University, Faisalabad. Prior to feeding trial, rats were acclimatized by giving basal diet for few days. During eight week feeding trial, temperature and relative humidity was maintained to 25 ± 2 °C and 55 ± 5%, respectively, with regular alteration in light/dark period consisting of 12 h. Central Government Ethical Committee Guidelines were strictly followed during the course of study (Approval no. FSD-16/M-234).

### 2.6. Diet and Dosage

For control group, the diet is composed of 66% corn starch, 10% corn oil, 10% cellulose, 10% protein contents, 3% minerals and 1% vitamin mixture. For experimental groups, skin, flesh, whole fruit powder of bitter melon in doses of 150 mg/kg and 300 mg/kg body weight was added. In this way, seven groups of rats were made consisting of twenty rats in each group. Each group is further split into normal rats and hyperglycemic rats. The normal rats were given diet without excessive sucrose while hyperglycemic rats were given 40% sucrose in diet to induce hyperglycemia and diabetes. The rats were given access to water and feed on ad libitum basis.

### 2.7. Testing

Each group was observed for water intake, feed intake and weight gain in 24 h period during the course of the study. The volume of urine was also noted by using metabolic cages in which rats were placed for 24 h and collected urine under a layer of toluene. The amount of reducing sugar in urine of rats was determined by 3,5-dintro salicylic acid method [17]. Creatinine was measured in blood and urine by Owen’s method [18]. Glomerular filtration rate (GFR) was assessed by following formula [19]:

$$\begin{array}{c} \mathrm{GFR}\left(\mathrm{ml}/\min\right)\\ =\frac{\mathrm{Urine}\;\mathrm{volume}\;\left(\mathrm{ml}\right)\times \mathrm{Urinary}\;\mathrm{Creatinine}\;\left(\mathrm{mg}/\mathrm{dl}\right)\times 1000\;\left(g\right)}{\mathrm{Body}\;\mathrm{weight}\;\left(g\right)\times \mathrm{Plasma}\;\mathrm{creatinine}\;\left(\mathrm{mg}/\mathrm{dl}\right)\times 1440\;\left(\min\right)} \end{array}$$

Blood samples were collected in EDTA coated tubes at 28 and 56 day of trial. Isolation of serum was done by centrifugation @ 4000 rpm for 6 min in centrifuge machine (Rotrofix 32-A Heltich, Westphalia, Germany). For biochemical evaluation, these samples of sera were kept through Microlab (Rendox Toerauta RX-Monza, County Monaghan, Republic of Ireland). The glucose and insulin level was calculated by procedure of Katz et al. [20] and Ahn et al. [21], respectively. The percent increase or decrease in biochemical traits of experimental groups was calculated in comparison to control groups. At the end of trial, the weight of the right and left kidneys were determined separately.

### 2.8. Statistical Analysis

The data was statistically analyzed using Statistical Package (Microsoft Excel 2016 and SPSS v20.). Level of significance was determined by ANOVA and LSD for comparison [22].

## 3. Results

### 3.1. Quantification of Charantin and Vicine

**Figure 1:**
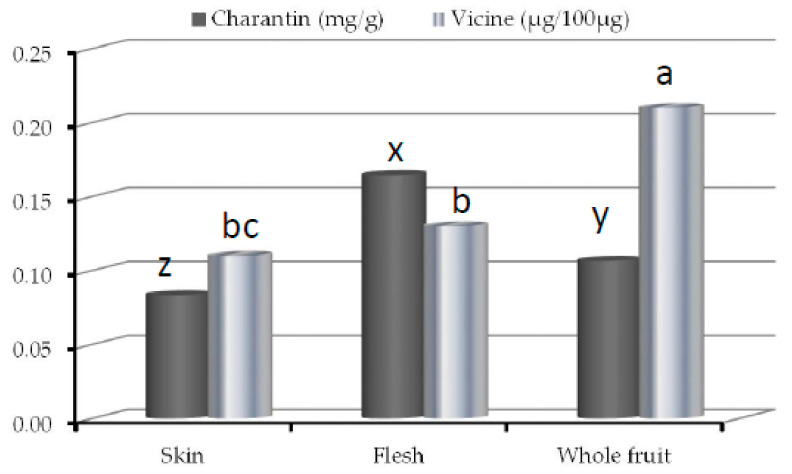
Charantin and vicine contents in skin, flesh and whole fruit of bitter melon. x,y,z indicated significant differences in charantin contents (*p* < 0.05); a,b,c indicated significant differences in vicine contents (*p* < 0.05).

The charantin and vicine contents (Figure 1) in different bitter melon fruit parts were determined through HPLC before administration of diet to Sprague Dawley rats. The total amount of charantin was found high in flesh part (0.16 ± 0.02 mg/g) than whole fruit (0.11 ± 0.02 mg/g) and skin (0.08 ± 0.01 mg/g). Meanwhile, whole fruit possessed high amount of vicine (0.210 ± 0.010 μg/100 μg) than flesh (0.131 ± 0.005 μg/100 μg) and skin (0.114 ± 0.006 μg/100 μg).

### 3.2. Feed and Water Intake

The diet containing bitter melon fruit skin, flesh and whole fruit at the doses of 150 and 300 mg/kg body weight increases the consumption of water in normal rats with highest increase was observed in rats fed with 300 mg/kg body weight of whole fruit (Table 1).

In hyperglycemic rats, consumption of water increased in control and significant reduction in water intake was observed by incorporation of bitter melon in diet in dose dependent manner. Whole fruit and flesh of bitter melon were found more effective than skin in preventing polydipsia conditions in hyperglycemic rats. In normal rats, supplementation of bitter gourd skin, flesh and whole fruit has resulted in reduction of feed intake compared to control while polyphagia condition was noticed in hyperglycemic control rats as they consumed an excessive quantity of diet compared to rats fed with bitter melon (Table 2).

### 3.3. Body Weight Gain

Normal control rats gained more weight with the passage of time than bitter melon fed rats (Table 3). In hyperglycemic control rats, body weight did not increase as much, while apprehensible body weight increase was observed in hyperglycemic rats fed with bitter melon. Increase in body weight was more pronounced in hyperglycemic rats fed with 300 mg/kg body weight of bitter melon than 150 mg/kg body weight.

### 3.4. Estimation of Urine and Reducing Sugar Excretion

The hyperglycemic rats produced more urine than the normal rats. This polyuria condition prevailed in hyperglycemic control group (Table 4). The dietary supplementation of bitter melon unveiled substantial decrease in urine excretion.

The amount of reducing sugar in excreted urine was negligible (in milligrams) in normal control and experimental rats (Table 5). The urine samples of hyperglycemic control rats showed large quantity of reducing sugar and decreased considerably in rats fed with diet containing skin, flesh and whole fruit of bitter melon. The most potent dose was 300 mg/kg body weight of the whole fruit of bitter melon in reducing glycosuria condition followed by flesh and skin.

### 3.5. Estimation of Kidney Weight

The assessment of renal hypertrophy was determined by observing the kidney weights in relation to 100 g body weight (Table 6). Normal rats showed no abnormal increase in kidney weight. During hyperglycemic conditions, increase in kidney weight is more obvious in control rats specifying renal hypertrophy. Bitter melon dietary supplementation significantly reduced this condition.

### 3.6. Estimation of Glomerular Filtration Rate

Significantly higher glomerular filtration rate (Table 7) was noticed in hyperglycemic control rats and regular bitter gourd consumption in these rats markedly influenced this trait to normalization.

### 3.7. Estimation of Blood Glucose

**Figure 2:**
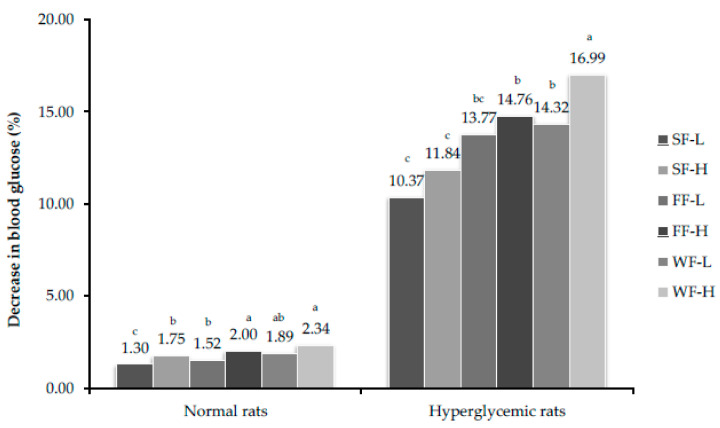
Percent decrease in glucose level after feeding bitter gourd on 28th day. Bars that do not share similar letters denote statistical significance (*p* < 0.05).

**Figure 3:**
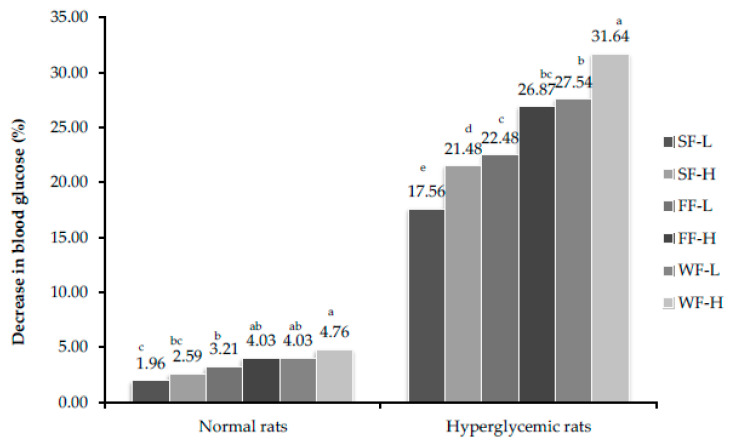
Percent decrease in glucose level after feeding bitter gourd on 56th day. Bars that do not share similar letters denote statistical significance (*p* < 0.05).

In normal rats, due to feeding of bitter melon, blood sugar level was maintained in experimental group of rats. In hyperglycemic rats, blood sugar level reduced significantly in experimental groups. The maximum reduction was observed in rats fed with whole fruit of bitter gourd (300 mg/kg body weight) in both 28th (Figure 2) and 56th (Figure 3) day samples.

### 3.8. Estimation of Insulin Concentration

**Figure 4:**
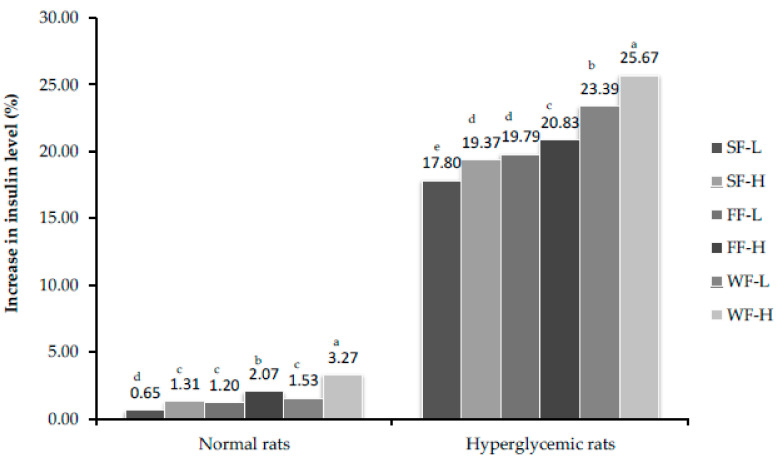
Percent increase in insulin level after feeding bitter gourd on 28th day. Bars that do not share similar letters denote statistical significance (*p* < 0.05).

**Figure 5:**
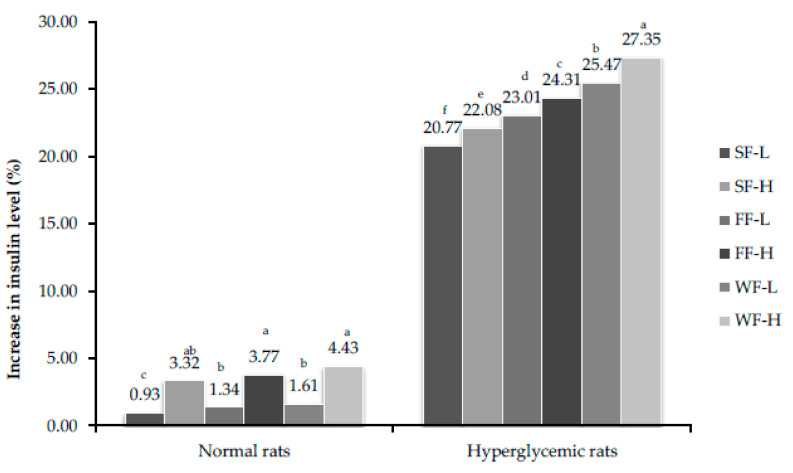
Percent increase in insulin level after feeding bitter gourd on 56th day. Bars that do not share similar letters denote statistical significance (*p* < 0.05).

Skin, flesh and whole fruit of bitter melon in diet induced a significant increase in insulin release from the pancreas of diabetic rats at both low (150 mg/kg body weight) and high (300 mg/kg body weight) concentrations in a dose dependent manner as compared to control values in normal and hyperglycemic rats. Skin fed rats showed a lesser effect on insulin release compared to flesh and whole fruit. Treatments of hyperglycemic rats with bitter melon for 4 and 8 weeks resulted in significant increase in insulin production (Figure 4 and Figure 5). This section may be divided by subheadings. It should provide a concise and precise description of the experimental results and their interpretation, as well as the experimental conclusions that can be drawn.

## 4. Discussion

There is an erratic approach in the literature regarding effectiveness of bitter melon on glucose control to make a decisive conclusion. The present, detailed study highlighted the effectiveness of skin, flesh and whole fruit containing seed as a dietary approach against development of diabetes and its treatment. The present results clearly revealed that bitter melon given at 300 mg/kg body weight improved the diabetic status to a reasonable extent. Bitter melon possessed high concentration of hypoglycemic agents like charantin and vicine, which are the mainstays in treatment of diabetes. Charantin and vicine contents were first observed in skin, flesh and whole fruit containing seeds before bio-evaluation. Pitipanapong et al. [15], Ham and Wang [23] and Desai et al. [24] reported variable concentration of charantin in this plant. Similarly, a higher amount of vicine was assessed in seeds than fruit and leaves by Zhang et al. [16]. The skin, flesh and whole fruit of bitter melon with known quantities of charantin and vicine were administered in daily diet of experimental rats.

Improvements in diabetic conditions were observed after giving bitter melon. The increase in water intake in experimental groups of normal rats is due to increase in metabolic rate and fatty acid metabolism after incorporation of bitter melon in diet. The excessive water consumption by hyperglycemic rats is primarily associated with pre-diabetic state and characteristic sign of onset of diabetes. This notion is favored by the findings of Parmar et al. [25], who reported high intake of water in hyperglycemic rats. In diabetic rats, intake of water gradually reduced after giving bitter melon [25].

Feed intake reduced to certain degree due to incorporation of bitter melon in diet. Chen et al. [26], Reyes et al. [27] and Huang et al. [28] also observed lesser feed intake in rats after adding bitter melon, but this impact is trivial to be considered or even in some cases have no impact on feed intake [29]. The gradual increase in body weight was observed in normal control, whereas that increase was lesser in diabetic control rats. The other diabetic groups of rat that fed on bitter melon, improvement in body weight was observed [30]. Bitter melon inclusions in the diet prevent the polyuria condition associated with diabetes. In hyperglycemic rats, urine sugar was very high and in agreement with previous studies [31]. Dietary supplementation of bitter melon minimizes sugar excretion in urine during diabetes. Increase in kidney size and malfunctioning is generally associated with diabetes. This increase is mainly due to increase in diameter and length of capillaries. In current study, bitter gourd supplementation has resulted in partial but significant decrease in ratio of kidney to body weight. Increase in glomerular filtration rate in hyper-functional kidney mostly occurs during the initial diabetic stage [32] and metabolic control for a long period is helpful in reducing kidney filtration during diabetes [33]. In our study, the glomerular filtration rate increased considerably in hyperglycemic control. The feeding of bitter melon to hyperglycemic rats showed significant reduction in glomerular filtration rate. The hypoglycemic ability of bitter gourd is discussed in different scientific explorations, advocating its use in various forms. The results regarding reduction in blood glucose level by consuming bitter melon are in accordance with the findings of Jafri et al. [30]. In their study, hyperglycemic rats showed a substantial decrease in glucose when fed with bitter melon powder. In another study, acetone extract of whole bitter melon fruit powder was used in doses of 25, 50 and 75 mg/100 g body weight and was observed to have significant blood glucose lowering ability (13.30 to 50%) in alloxan diabetic albino rats [34]. On the other hand, some studies reported contradictory results of bitter melon intake with no positive effect in lowering blood glucose level and diabetic conditions [12,13].

The hypoglycemic ability of bitter melon is due to presence of bioactive molecules that play a pivotal role in many physiological, pharmacological and biochemical processes [35]. The possible factors involved in reducing blood glucose level by bitter melon consumption include skeletal and peripheral muscles stimulation to enhance glucose utilization [36,37], preventing uptake of glucose from intestine [38,39,40], maintaining enzymatic activities related to glucose metabolism [41], restoring stability of pancreatic β-cells and increasing their functionality [42]. Bitter melon contains charantin and vicine, which are collectively found more effective in diabetes management as charantin is a typical cucurbitane-type triterpenoid in *M. charantia* and is a potential substance with antidiabetic properties. Charantin could be used to treat diabetes and can potentially replace treatment. It is a mixture of two compounds, namely, sitosteryl glucoside and stigmasteryl glucoside [43].

In the present study, bitter melon supplemented feed had a positive effect on pancreatic cells to enhance insulin secretion and maintenance of serum insulin level [44,45]. This increase in insulin production might be due to β-cells structural and functional stability after consuming diet supplemented with bitter melon [34,43] or increasing the number of β-cells in Islets of Langerhans [44]. According to Xiang et al. [46], certain chemical constituents in bitter melon operated as a growth factor for pancreatic β-cells. The presence of bioactive moieties that act as hypoglycemic agents including charantin and vicine are spontaneously involved in insulin production or possess insulin-like activity. The chemical constituents present in bitter melon help in minimizing oxidative damage by neutralization of free radicals activity and performing prompt actions to control the death of β-cells. From the above discussion, it is deduced that all parts of fruits of bitter melon are significantly effectual against diabetes and 300 mg/kg body weight of whole fruit was the most potent dose and can be preferred for use in hyperglycemic conditions and diabetes prophylaxis and treatment.

## 5. Conclusions

It is concluded that charantin is present in a large quantity in flesh parts and vicine is mainly concentrated in the whole fruit containing seeds. Current research intervention suggested that individual phytochemical like charantin or vicine is less effective in diabetes management. The complex interaction of these hypoglycemic agents of bitter melon can play a more operative role in delaying the pathogenesis of diabetes mellitus. However, the clinical studies following the double blind randomized controlled trials on human subjects must be conducted to warrant their use in humans.

## Figures and Tables

**Table 1:** Impact of bitter melon supplementation on water intake of rats.

Groups	Water Intake (mL/24 h)			
	28th day		56th day	
	Normal Rats	Hyperglycemic Rats	Normal Rats	Hyperglycemic Rats
Control	23.7 ± 1.15 ^d^	29.35 ± 2.15 ^a^	27.9 ± 2.12 ^d^	36.65 ± 2.05 ^a^
SF-L	24.1 ± 1.34 ^c^	25.94 ± 1.49 ^b^	28.1 ± 1.77 ^c,d^	31.02 ± 2.34 ^b^
SF-H	24.3 ± 1.40 ^c^	24.85 ± 1.46 ^c^	28.4 ± 2.15 ^c^	29.01 ± 2.17 ^d^
FF-L	24.4 ± 1.88 ^b,c^	25.03 ± 2.11 ^b^	28.3 ± 1.63 ^c^	30.85 ± 1.69 ^c^
FF-H	24.7 ± 1.35 ^b^	24.07 ± 1.75 ^d^	28.6 ± 2.19 ^b^	28.79 ± 1.56 ^e^
WF-L	24.8 ± 2.01 ^a,b^	24.91 ± 1.68 ^b^	28.7 ± 2.03 ^b^	30.71 ± 2.62 ^c^
WF-H	25.2 ± 1.48 ^a^	23.86 ± 1.33 ^d^	29.00 ± 1.59 ^a^	28.63 ± 2.22 ^e^

SF—Skin fed rats; FF—Flesh fed rats; WF- Whole fruit fed rats; L—Lower amount of bitter melon in experimental diet (150 mg/kg body weight), H—High amount of bitter melon in experimental diet (300 mg/kg body weight); Values are mean ± SD of ten rats; Superscript letters within the same column indicate significant (*p* < 0.05) differences.

**Table 2:** Impact of bitter melon supplementation on feed intake of rats.

Groups	Feed Intake (g)			
	28th day		56th day	
	Normal Rats	Hyperglycemic Rats	Normal Rats	Hyperglycemic Rats
Control	15.1 ± 1.12 ^a^	17.03 ± 1.78 ^a^	21.2 ± 2.49 ^a^	23.22 ± 1.33 ^a^
SF-L	14.6 ± 0.76 ^b^	14.96 ± 1.39 ^b^	20.5 ± 2.03 ^b^	20.94 ± 1.11 ^b^
SF-H	14.3 ± 1.38 ^b^	13.80 ± 1.01 ^c^	20.1 ± 1.98 ^b^	19.78 ± 1.04 ^c^
FF-L	14.3 ± 1.22 ^b^	14.94 ± 0.78 ^b^	20.4 ± 1.62 ^b^	20.87 ± 0.92 ^b^
FF-H	14.1 ± 0.94 ^b,c^	13.76 ± 1.39 ^c^	19.8 ± 0.99 ^b,c^	19.45 ± 1.17 ^c^
WF-L	13.9 ± 0.45 ^c^	14.82 ± 1.54 ^b^	19.9 ± 1.07 ^b,c^	20.73 ± 1.43 ^b^
WF-H	13.7 ± 0.88 ^c^	13.64 ± 1.44 ^c^	19.6 ± 0.69 ^c^	19.36 ± 1.36 ^c^

Abbreviations and description as in Table 1.

**Table 3:** Impact of bitter melon supplementation on body weight of rats.

Groups	Body Weight (G)			
	28th Day		56th Day	
	Normal Rats	Hyperglycemic Rats	Normal Rats	Hyperglycemic Rats
Control	158.66 ± 3.01 ^a^	145.55 ± 2.75 ^c^	215.90 ± 4.22 ^a^	181.34 ± 2.61 ^c^
SF-L	155.91 ± 2.77 ^b^	151.18 ± 2.04 ^b^	208.67 ± 4.06 ^b^	203.66 ± 3.59 ^b^
SF-H	154.75 ± 3.52 ^c^	151.66 ± 1.89 ^a,b^	208.40 ± 2.88 ^b^	205.33 ± 3.11 ^a^
FF-L	154.62 ± 1.95 ^c^	151.43 ± 2.09 ^b^	208.33 ± 2.54 ^b^	204.50 ± 3.49 ^b^
FF-H	154.34 ± 2.67 ^c^	152.57 ± 2.67 ^a^	208.13 ± 1.97 ^b^	206.56 ± 3.34 ^a^
WF-L	154.32 ± 3.00 ^c^	151.71 ± 0.95 ^b^	208.26 ± 2.42 ^b^	204.66 ± 2.68 ^b^
WF-H	153.68 ± 2.18 ^d^	152.83 ± 1.90 ^a^	208.01 ± 2.97 ^b^	206.60 ± 2.36 ^a^

Abbreviations and description as in Table 1.

**Table 4:** Impact of bitter melon supplementation on urine volume of rats.

Groups	Urine Volume (mL/24 h)			
	28th Day		56th Day	
	Normal Rats	Hyperglycemic Rats	Normal Rats	Hyperglycemic Rats
Control	14.3 ± 2.23 ^a^	20.1 ± 1.01 ^a^	18.3 ± 1.13 ^a^	26.8 ± 2.85 ^a^
SF-L	13.7 ± 1.72 ^b^	16.5 ± 2.19 ^b^	17.6 ± 1.31 ^b^	21.3 ± 3.03 ^b^
SF-H	13.6 ± 1.63 ^b^	15.8 ± 1.43 ^b^	17.5 ± 0.99 ^b^	20.65 ± 1.26 ^b^
FF-L	13.3 ± 0.76 ^b,c^	15.7 ± 1.29 ^b^	17.3 ± 1.84 ^b^	20.4 ± 1.64 ^b^
FF-H	13.3 ± 1.33 ^b,c^	14.9 ± 2.07 ^b^	16.9 ± 1.22 ^b,c^	20.2 ± 2.13 ^b,c^
WF-L	13.2 ± 0.89 ^c^	15.2 ± 0.96 ^c^	16.8 ± 1.71 ^c^	19.9 ± 0.78 ^c^
WF-H	13.0 ± 2.13 ^c^	14.7 ± 1.56 ^d^	16.7 ± 2.17 ^c^	19.7 ± 1.16 ^c^

Abbreviations and description as in Table 1.

**Table 5:** Impact of bitter melon supplementation on reducing sugar in urine.

Groups	Reducing Sugar in Urine (G)			
	28th Day		56th Day	
	Normal Rats	Hyperglycemic Rats	Normal Rats	Hyperglycemic Rats
Control	0.02 ± 0.00 ^a^	2.91 ± 0.32 ^a^	0.02 ± 0.01 ^a^	4.32 ± 0.75 ^a^
SF-L	0.03 ± 0.02 ^a^	2.16 ± 0.11 ^b^	0.03 ± 0.02 ^a^	2.18 ± 0.42 ^b^
SF-H	0.02 ± 0.01 ^a^	1.86 ± 0.21 ^c^	0.02 ± 0.01 ^a^	1.96 ± 0.38 ^b^
FF-L	0.05 ± 0.02 ^a^	1.99 ± 0.36 ^c^	0.04 ± 0.02 ^a^	2.02 ± 0.33 ^b^
FF-H	0.02 ± 0.00 ^a^	1.58 ± 0.58 ^c^	0.02 ± 0.01 ^a^	1.74 ± 0.27 ^c^
WF-L	0.02 ± 0.01 ^a^	1.87 ± 0.13 ^c^	0.03 ± 0.00 ^a^	1.89 ± 0.14 ^b^
WF-H	0.03 ± 0.01 ^a^	1.43 ± 0.19 ^d^	0.02 ± 0.00 ^a^	1.63 ± 0.36 ^c^

Abbreviations and description as in Table 1.

**Table 6:** Impact of bitter melon supplementation on kidney weight.

Groups	Kidney Weight (G) in Normal Rats		Kidney Weight (G) in Hyperglycemic Rats	
	Right	Left	Right	Left
Control	0.69 ± 0.04 ^a^	0.68 ± 0.01 ^a^	0.89 ± 0.06 ^a^	0.87 ± 0.03 ^a^
SF-L	0.64 ± 0.03 ^b^	0.63 ± 0.02 ^b^	0.78 ± 0.03 ^b^	0.78 ± 0.02 ^b^
SF-H	0.63 ± 0.02 ^b^	0.62 ± 0.01 ^b^	0.76 ± 0.04 ^c^	0.77 ± 0.02 ^b^
FF-L	0.64 ± 0.02 ^b^	0.63 ± 0.03 ^b^	0.76 ± 0.03 ^c^	0.75 ± 0.04 ^c^
FF-H	0.62 ± 0.01 ^c^	0.62 ± 0.04 ^b^	0.74 ± 0.03 ^d^	0.72 ± 0.03 ^d^
WF-L	0.64 ± 0.03 ^b^	0.62 ± 0.02 ^b^	0.69 ± 0.02 ^e^	0.70 ± 0.01 ^d,e^
WF-H	0.62 ± 0.02 ^c^	0.62 ± 0.02 ^b^	0.68 ± 0.02 ^e^	0.68 ± 0.02 ^e^

Abbreviations and description as in Table 1.

**Table 7:** Impact of bitter melon supplementation on glomerular filtration rate of rats.

Groups	Glomerular Filtration Rate (mL/min)	
	Normal Rats	Hyperglycemic Rats
Control	1.13 ± 0.13 ^a^	3.32 ± 0.33 ^a^
SF-L	1.06 ± 0.09 ^b^	2.76 ± 0.27 ^b^
SF-H	1.04 ± 0.11 ^b^	2.70 ± 0.28 ^b^
FF-L	1.04 ± 0.16 ^b^	2.75 ± 0.22 ^b^
FF-H	1.03 ± 0.07 ^b,c^	2.63 ± 0.31 ^b^
WF-L	1.04 ± 0.09 ^b^	2.45 ± 0.21 ^c^
WF-H	1.01 ± 0.14 ^c^	2.33 ± 0.14 ^c^

Abbreviations and description as in Table 1.

## Data Availability

All the data produced here is available and can produced when required.
